# Supplementary material for: Tumor-Associated Macrophages Promote Brain Metastasis
Source: bioRxiv. 2026 Jun 30:2026.06.29.735286. Preprint. [Version 1] doi: 10.64898/2026.06.29.735286 (PMC13345047; doi:10.64898/2026.06.29.735286)
Supplement: Supplement 1 [file NIHPP2026.06.29.735286v1-supplement-1.pdf]

105 and is also made available for use under a CC0 license.

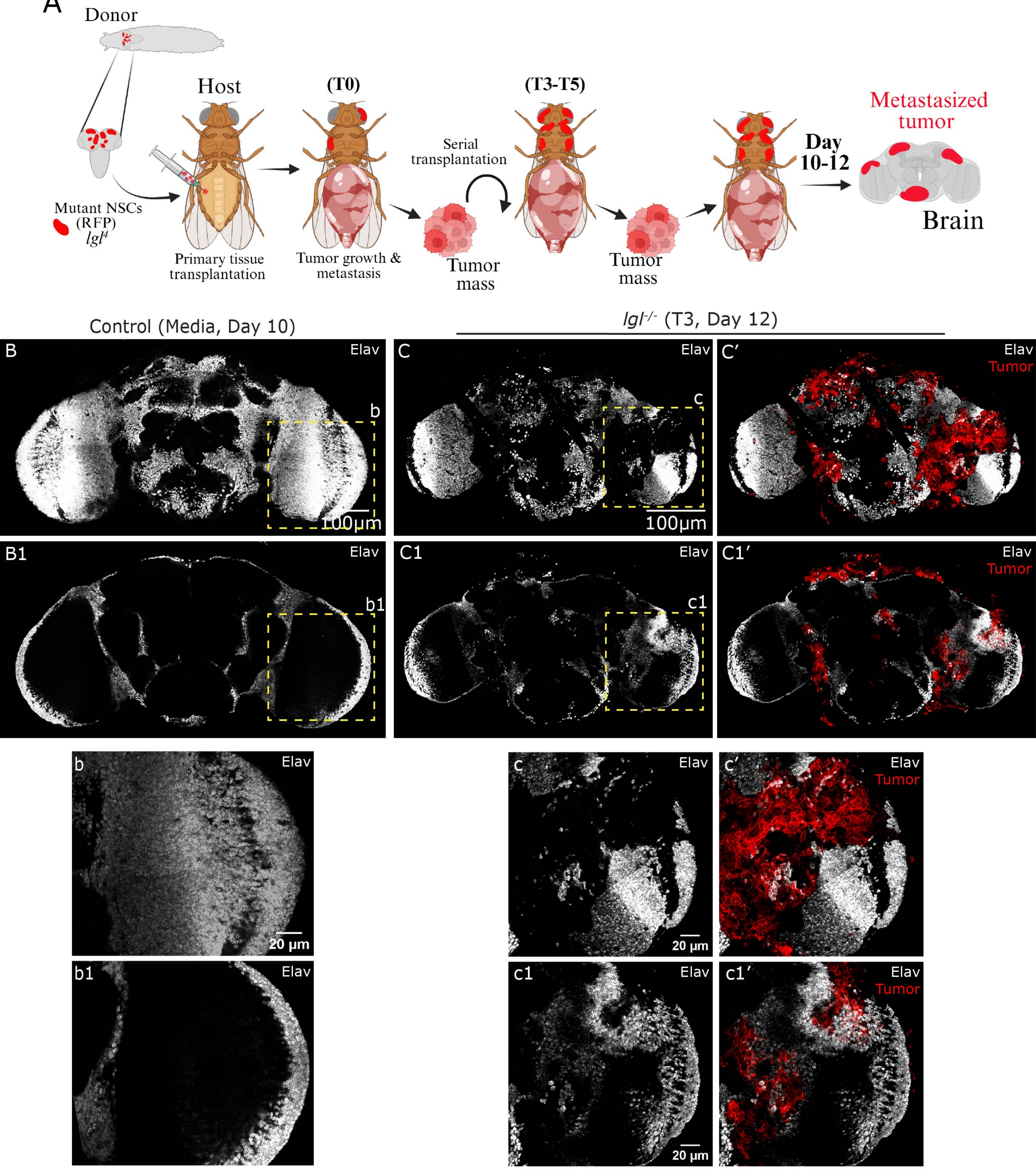

Khan and Rusan, Figure S1

bioRxiv preprint doi: https://doi.org/10.64898/2026.06.29.735286; this version posted June 30, 2026. The copyright holder for this preprint (which was not certified by peer review) is the author/funder. This article is a US Government work. It is not subject to copyright under 17 USC 105 and is also made available for use under a CC0 license.

Figure S1 *lgl*$^{-/-}$ tumors deform and disrupt neuronal tissue organization in the *Drosophila* brain. (A) Schematic illustration of the tumor transplantation model. *lgl*$^{-/-}$ tumor cells are dissected and injected into the abdomen of host *Drosophila*. Tumors grow progressively within the host and ultimately metastasize to the brain. **(B-B1)** Representative confocal z-stack images of a control (Media, Day 10) brain at two z-levels showing Elav-positive neurons (grey scale). **(b-b1)** High-magnification images of the boxed regions in **B** and **B1**, respectively, showing Elav distribution in control brains. **(C-C')** Representative confocal z-stack images of an *lgl*$^{-/-}$ brain (T3, Day 12) at the top z-level showing Elav (grey) alone (**C**) and merged with tumor cells (red) (**C'**). **(C1-C1')** Mid z-level images of the same *lgl*$^{-/-}$ brain showing Elav alone (**C1**) and merged with tumor (red) (**C1'**). **(c-c')** High-magnification images of the boxed region in **C** showing Elav (grey) alone and merged with tumor cells (red), respectively, illustrating tumor disrupting the neuronal cell cortex. **(c'-c1')** High-magnification images of the boxed region in **C1** showing Elav (grey) alone and merged with tumor (red) at a deeper z-section.

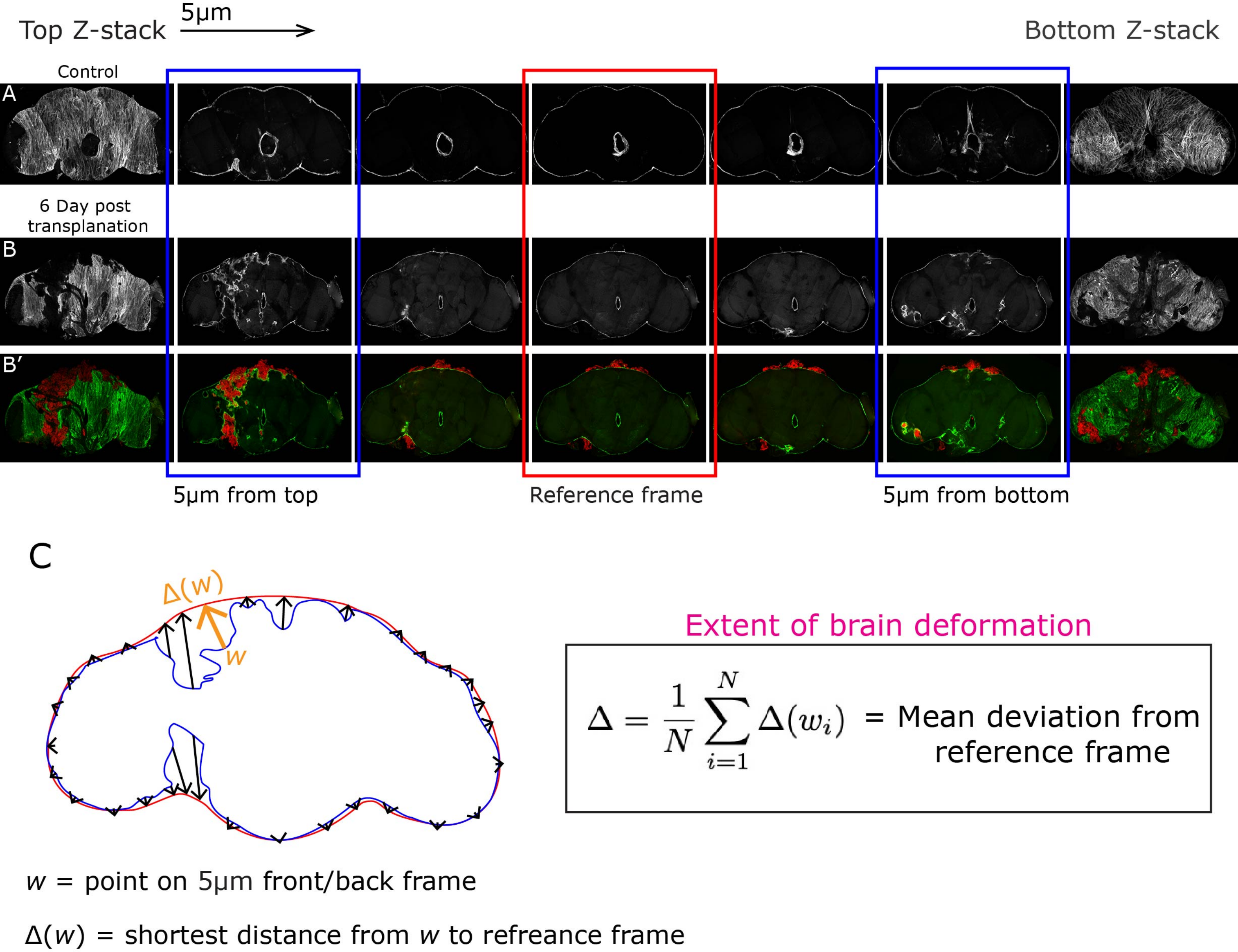

Khan and Rusan, Figure Suppl 2

bioRxiv preprint doi: https://doi.org/10.64898/2026.06.29.735286; this version posted June 30, 2026. The copyright holder for this preprint (which was not certified by peer review) is the author/funder. This article is a US Government work. It is not subject to copyright under 17 USC 105 and is also made available for use under a CC0 license.

Figure S2 Schematic of the brain deformation quantification pipeline using serial confocal z-stack. (A) Representative serial confocal z-stack images spanning from the top to the bottom z-plane of a *Drosophila* brain, showing brain outline marker PNG-Gal4 driving mCD8-GFP. The central image highlighted by the red box denotes the reference frame (middle z-section) used as the fixed anatomical reference point. Blue boxes indicate the experimental frames at the top and bottom z-sections used for deformation comparison. **(B-B')** Corresponding serial z-stack images of an *lgl*$^{-/-}$ tumor-bearing brain shown in **B**, and a merged view of PNG glia (green) and tumor cells (red) in **B'**, across the same z-planes. The red-boxed central image represents the reference frame. Blue boxes highlight the top and bottom experimental frames. **(C)** Schematic diagram illustrating the method used to quantify the extent of brain deformation. The brain outline from the reference frame (middle z-section, red) is superimposed onto the outline from an experimental frame (blue). w represent point on brain outline on experimental frame, and $\Delta(w)$ denotes the deviation of the experimental frame outline from the reference, used as a measure of deformation magnitude (see methods).

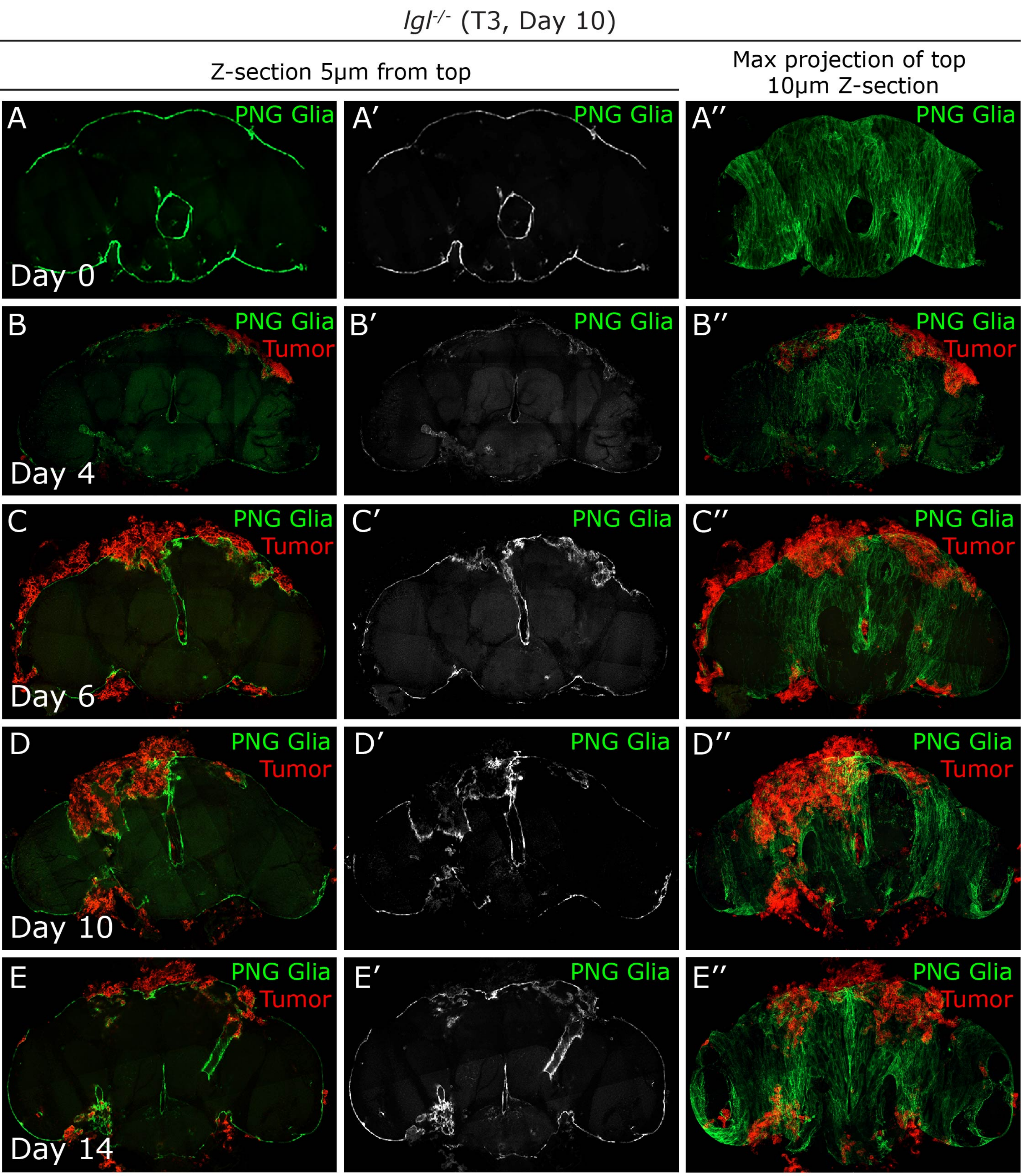

Khan and Rusan, Figure Suppl 3

bioRxiv preprint doi: https://doi.org/10.64898/2026.06.29.735286; this version posted June 30, 2026. The copyright holder for this preprint (which was not certified by peer review) is the author/funder. This article is a US Government work. It is not subject to copyright under 17 USC 105 and is also made available for use under a CC0 license.

Figure S3 Progressive disruption of brain surface by *lgl*$^{-/-}$ tumors over days. Representative confocal images brain showing PNG Glia (green) −5 μm z-section from the top, and max projection of top 10 μm z-sections at Day 0 **(A-A'')**, Day 4 **(B-B'')**, Day 6 **(C-C")**, Day 10 **(D-D")** and Day 14 **(E-E")** after T3-*lgl*$^{-/-}$ tumor (red) injection.

bioRxiv preprint doi: https://doi.org/10.64898/2026.06.29.735286; this version posted June 30, 2026. The copyright ho
(which was not certified by peer review) is the author/funder. This article is a US Government work. It is not subject to co
105 and is also ma[illegible] for use [illegible] CC0 license.

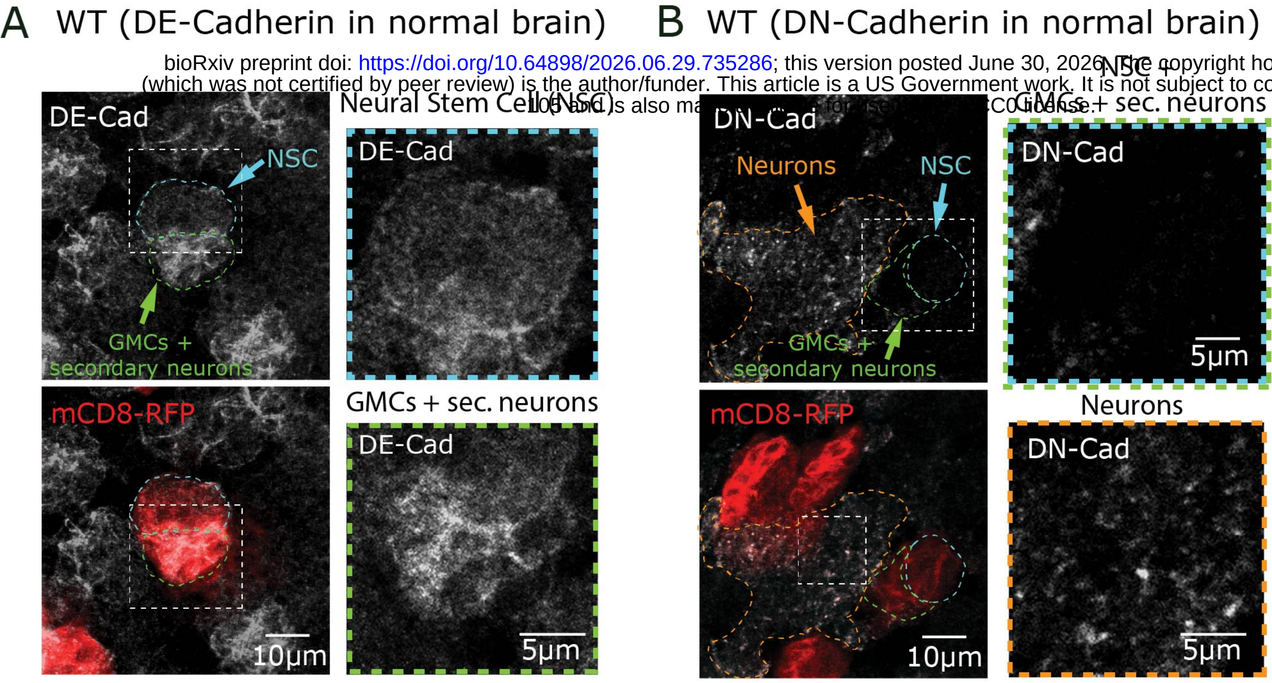

Khan and Rusan, Figure S4

bioRxiv preprint doi: https://doi.org/10.64898/2026.06.29.735286; this version posted June 30, 2026. The copyright holder for this preprint (which was not certified by peer review) is the author/funder. This article is a US Government work. It is not subject to copyright under 17 USC 105 and is also made available for use under a CC0 license.

Figure S4 DE-Cadherin and DN-Cadherin localization in wild-type brains cells. (A) Representative confocal images of a wild-type (WT) brain stained for DE-Cad (grey scale) with MARCM clone (mCD8-RFP, red) labeling neural stem cell lineage. Image showing DE-Cad distribution with a cyan outline indicating a neural stem cell (NSC, cyan arrow) and a green outline indicating ganglion mother cells (GMCs) and secondary neurons (GMCs + sec. neurons, green arrow). High-magnification insets showing DE-Cad localization in the NSC (cyan box) and GMC + secondary neuron region (green box). **(B)** Representative confocal images of a WT brain stained for DN-Cad (grey). Image showing DN-Cad distribution with an orange outline indicating neurons (orange arrow), a cyan + green outline indicating an NSC + GMCs (cyan + green arrow). High-magnification insets showing DN-Cad localization in the NSC + GMCs (cyan +green box) and secondary neuron region (orange box).

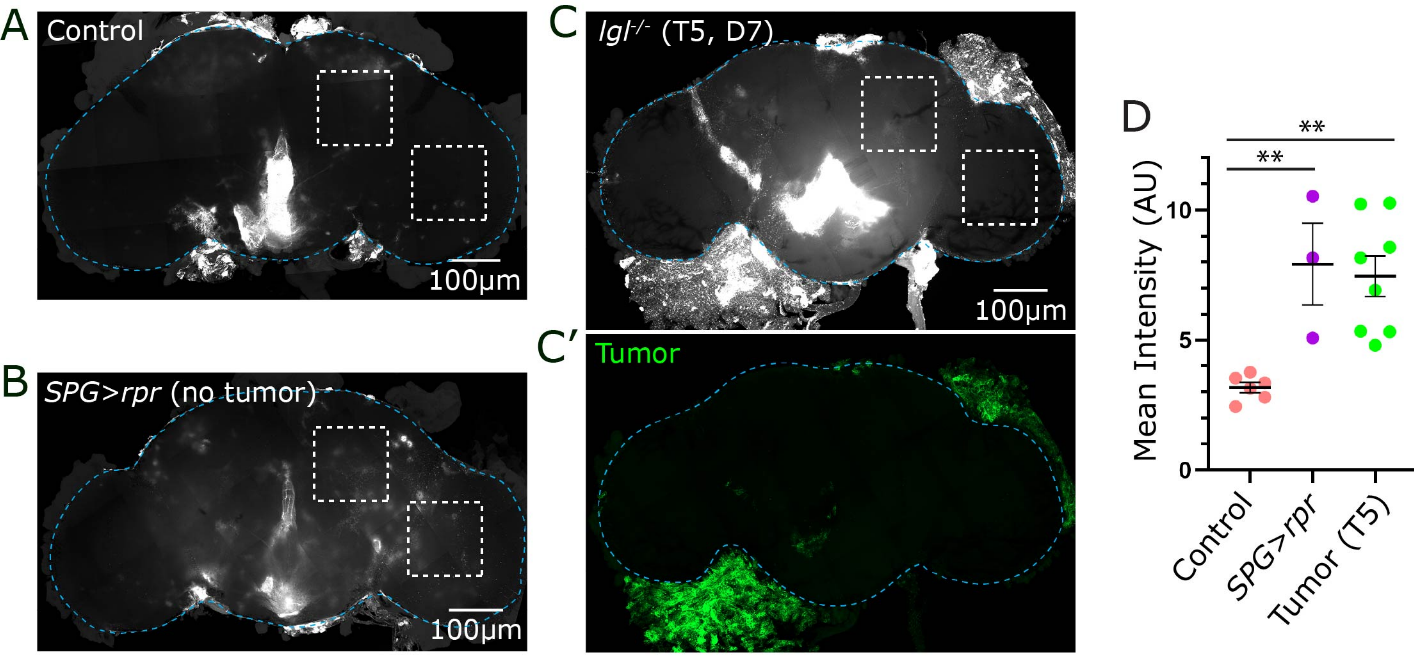

Khan and Rusan, Figure S5

bioRxiv preprint doi: https://doi.org/10.64898/2026.06.29.735286; this version posted June 30, 2026. The copyright holder for this preprint (which was not certified by peer review) is the author/funder. This article is a US Government work. It is not subject to copyright under 17 USC 105 and is also made available for use under a CC0 license.

Figure S5 *lgl*$^{-/-}$ tumor metastasis compromises functional integrity of the BBB. (A) Representative confocal image of a control brain showing dextran signal. **(B)** Representative confocal image showing dextran signal of a *SPG>rpr* brain (no tumor), in which subperineurial glia are genetically ablated via expression of the pro-apoptotic gene *rpr* under the *SPG*-Gal4 driver. **(C-C')** Representative confocal images of an *lgl*$^{-/-}$ tumor-bearing brain (T5, Day 7), **C** shows dextran signal and **C'** shows the corresponding tumor channel (green). White dashed boxes in (A-C) indicate ROIs used for mean intensity quantification. **(D)** Quantification of mean fluorescence intensity (arbitrary units, AU) measured within the ROIs shown in (A-C) for control, *SPG>rpr* no-tumor, and *lgl*$^{-/-}$ tumor T5. Data are presented as mean ± SEM, one-way ANOVA with Tukey's multiple comparisons test was used. $P < 0.05$ was considered statistically significant. Significance levels are indicated as follows: ****$P < 0.0001$, ***$P < 0.001$, **$P < 0.01$, and *$P < 0.05$.

bioRxiv preprint doi: https://doi.org/10.64898/2026.06.29.735286; this version posted June 30, 2026. The copyright holder for this preprint (which was not certified by peer review) is the author/funder. This article is a US Government work. It is not subject to copyright under 17 USC 105 and is also made available for use under a CC0 license.

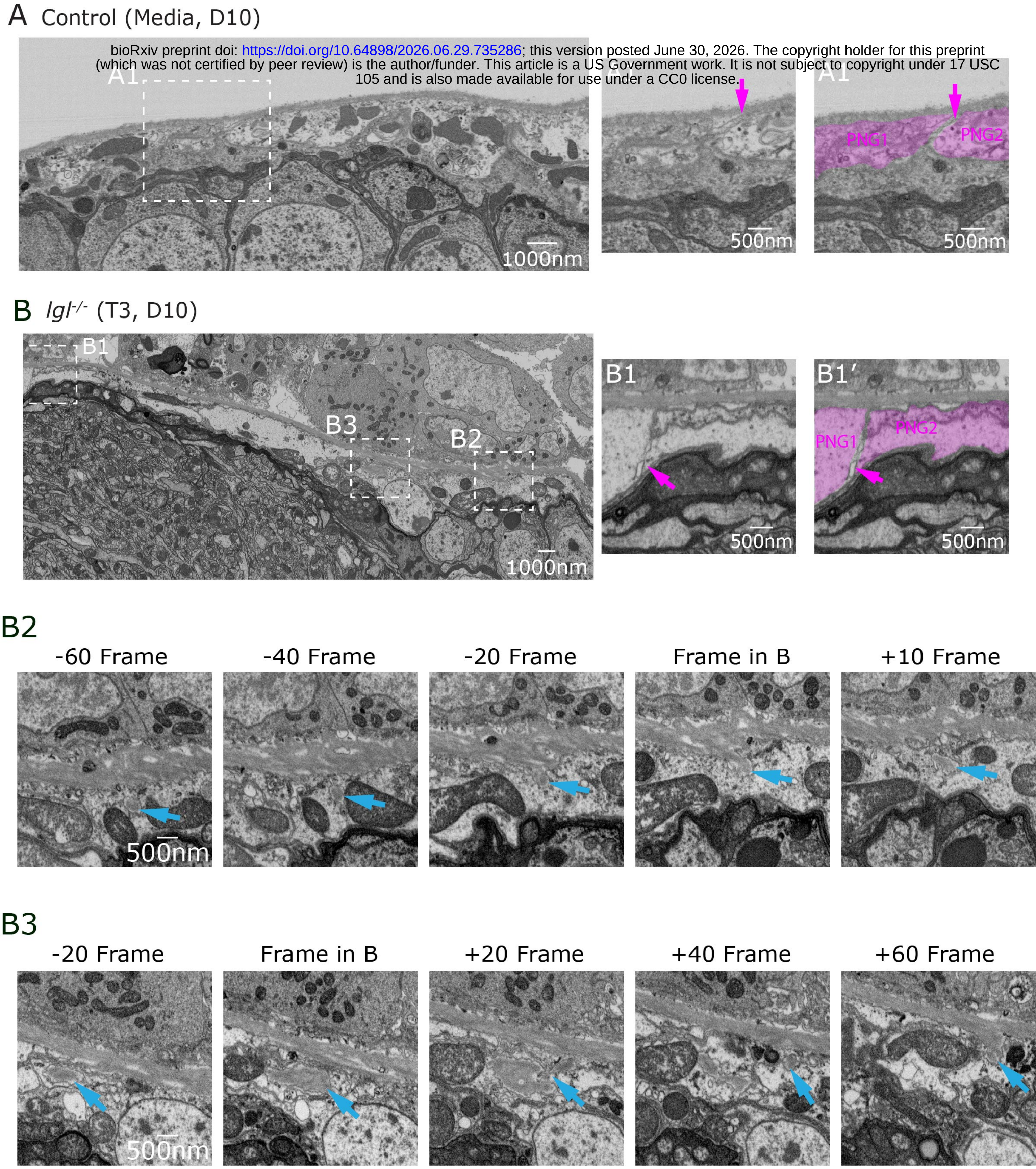

Khan and Rusan, Figure S6

bioRxiv preprint doi: https://doi.org/10.64898/2026.06.29.735286; this version posted June 30, 2026. The copyright holder for this preprint (which was not certified by peer review) is the author/funder. This article is a US Government work. It is not subject to copyright under 17 USC 105 and is also made available for use under a CC0 license.

Figure S6 Ultrastructural analysis of the BBB at metastatic site. (A) FIB-SEM overview image of a control (Media, Day 10) brain showing the intact BBB in cross-section. **(A1-A1')** High-magnification FIB-SEM image of the boxed region in A showing the normal ultrastructural organization of the PNG cells, the magenta arrow indicates intact PNG-PNG contact. **(B)** FIB-SEM overview image of an *lgl*$^{-/-}$ brain (Transplant 3, Day 10). **(B1-B1')** High-magnification FIB-SEM image of the boxed region in B the magenta arrow indicates loss of PNG-PNG contact. **(B2)** Serial FIB-SEM sections through the region boxed as B2 in B, shown at frames −60, −40, −20, the reference frame (Frame in B), and +10, spanning, blue arrows through consecutive sections marks vesicle containing ECM material in continuity with BM. **(B3)** Serial FIB-SEM section series through the same region at frames −20, the reference frame (Frame in B), +20, +40, and +60, blue arrows through consecutive sections marks vesicle containing ECM material in continuity with BM.

(which was not certified by peer review) is the author/funder. This article is a US Government work. It is not subject to cop
105 and is also made available for use under a CC0 license.

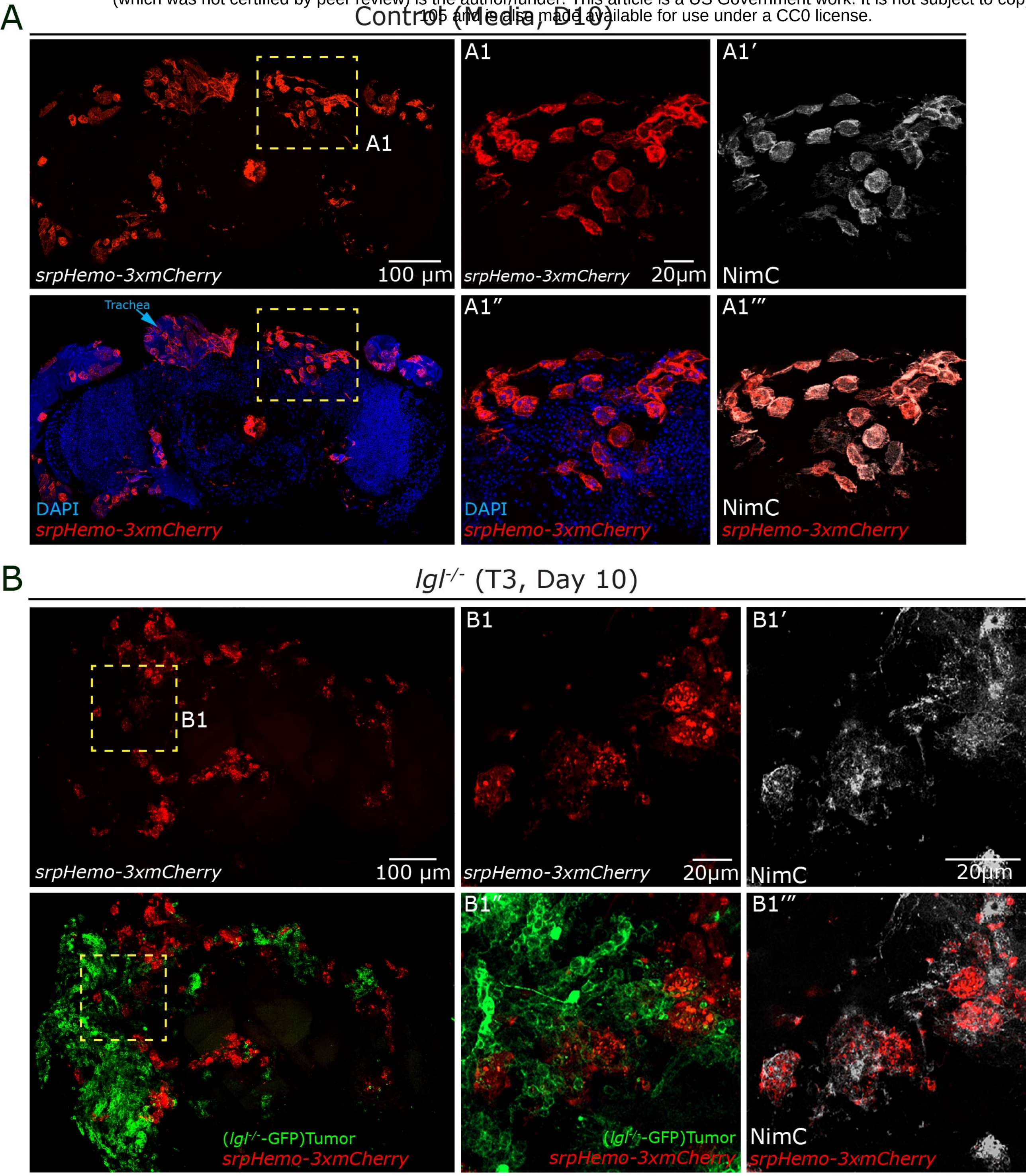

Khan and Rusan, Figure S7

bioRxiv preprint doi: https://doi.org/10.64898/2026.06.29.735286; this version posted June 30, 2026. The copyright holder for this preprint (which was not certified by peer review) is the author/funder. This article is a US Government work. It is not subject to copyright under 17 USC 105 and is also made available for use under a CC0 license.

Figure S7 *lgl*$^{-/-}$ tumors recruit activated macrophages. (A) Representative images of a control (Media, Day 10) brain showing hemocytes labeled with *srpHemo-3xmCherry* (red). **(A1)** High-magnification image of the boxed region showing *srpHemo-3xmCherry*-positive hemocytes (red). **(A1')** Corresponding NimC channel (grey scale) confirming hemocyte identity in the same region. **(A1")** High-magnification merged image showing DAPI (blue) and *srpHemo-3xmCherry* (red) hemocytes. **(A1"')** Merged image of NimC (white) and *srpHemo-3xmCherry* (red) confirming co-labeling of hemocytes by both markers in control brains. **(B)** Representative confocal images of an *lgl*$^{-/-}$ brain (T3, Day 10). **(B1)** High-magnification image showing *srpHemo-3xmCherry*-positive hemocytes (red) in the tumor-proximal region. **(B1')** Corresponding NimC channel (grey scale) showing hemocyte distribution in the same region, with enlarged and irregularly shaped cells consistent with an activated plasmatocyte morphology. **(B1")** High-magnification merged image of *lgl*$^{-/-}$ -GFP tumor cells (green) and *srpHemo-3xmCherry* hemocytes (red) showing hemocytes closely associated with tumor cells. **(B1"')** Merged image of NimC (white) and *srpHemo-3xmCherry* (red) confirming co-labeling of hemocytes by both markers in tumor-bearing brains.

bioRxiv preprint doi: https://doi.org/10.64898/2026.06.29.735286; this version posted June 30, 2026. The copyright holder for this preprint (which was not certified by peer review) is the author/funder. This article is a US Government work. It is not subject to copyright under 17 USC 105 and is also made available for use under a CC0 license.

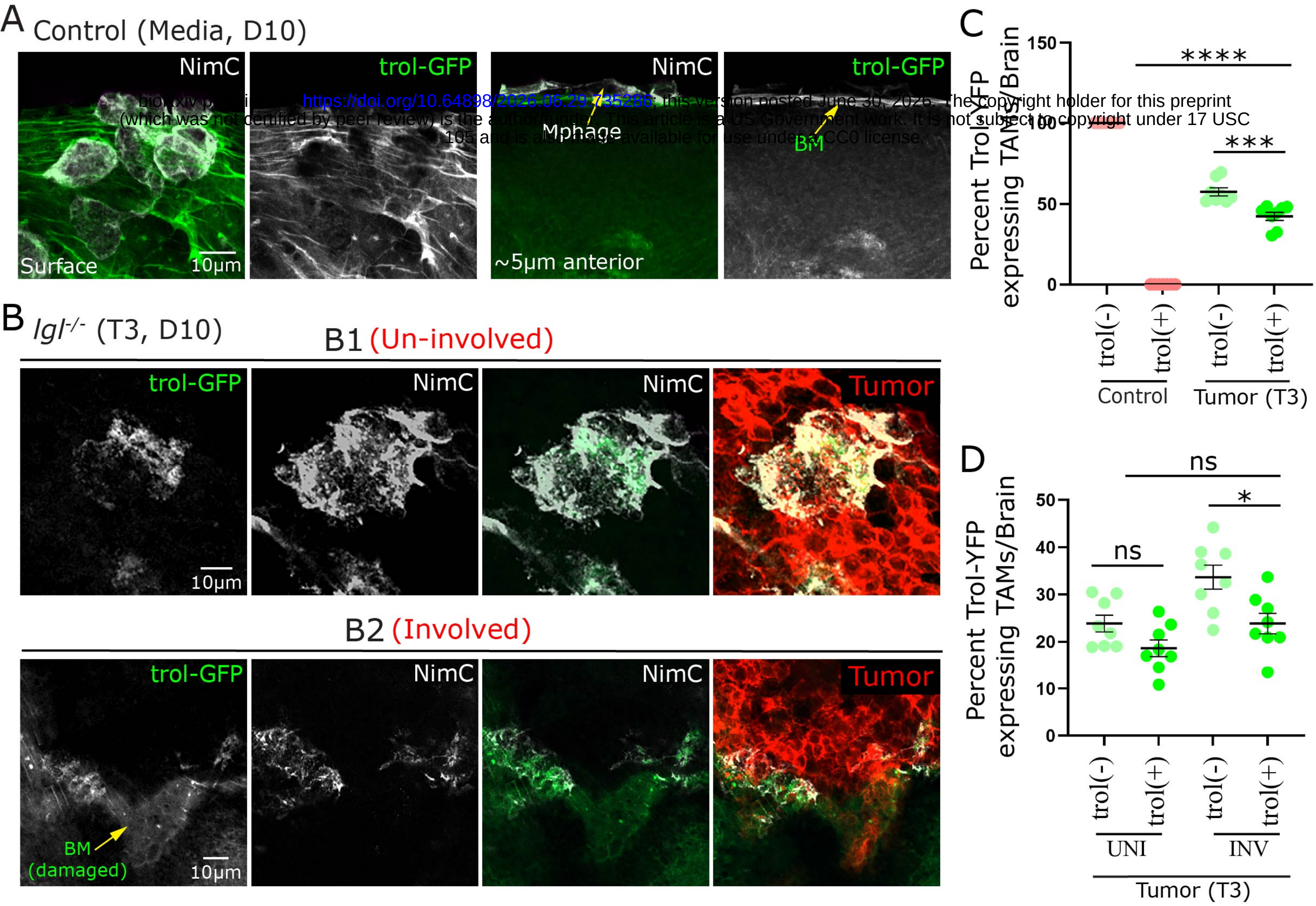

Khan and Rusan, Figure S8

bioRxiv preprint doi: https://doi.org/10.64898/2026.06.29.735286; this version posted June 30, 2026. The copyright holder for this preprint (which was not certified by peer review) is the author/funder. This article is a US Government work. It is not subject to copyright under 17 USC 105 and is also made available for use under a CC0 license.

Figure S8 TAMs positive Perlecan/Trol are uniformly distributed in tumor-involved regions of *lgl*-deficient brains. (A) Representative confocal images of a control (Media, Day 10) brain showing hemocyte-associated Trol expression. Left two panels, surface view showing NimC-positive hemocytes (grey scale) and *trol-GFP* signal (green) at the brain surface. Right panels, Z-section approximately 5 µm anterior to the surface showing a NimC-positive hemocyte (Mphage, yellow arrow) and *trol-GFP* signal (green) localized along the basement membrane (BM, yellow arrow). In control brains, *trol-GFP* expression is restricted to the BM with minimal hemocyte-associated signal. **(B)** Representative confocal images of an *lgl*$^{-/-}$ brain (Transplant 3, Day 10) showing Trol expression in TAMs across two distinct regions. **(B1)** (Un-involved region) High-magnification images showing *trol-GFP* (grayscale, left), NimC (grayscale (white, second panel), NimC in green (green, third panel), and tumor cells (red, right) in a region without direct tumor contact. **(B2)** (Involved region), High-magnification images showing *trol-GFP* (grayscale, left), NimC (grayscale (white, second panel), NimC in green (green, third panel), and tumor cells (red, right) in a region with direct tumor contact. **(C)** Quantification of the percentage of *trol-GFP*-expressing tumor-associated macrophages (TAMs) per brain in control and *lgl*$^{-/-}$ T3 transplanted, subdivided by *trol* expression status [trol(−) and trol(+)]. **(D)** Quantification of the percentage of *trol-GFP*-expressing TAMs per brain in un-involved (UNI) and tumor-involved (INV) regions of Tumor T3 brains, subdivided by trol expression status [trol(−) and trol(+)]. Data are presented as mean ± SEM, one-way ANOVA with Tukey's multiple comparisons test was used. $P < 0.05$ was considered statistically significant. Significance levels are indicated as follows: ****$P < 0.0001$, ***$P < 0.001$, **$P < 0.01$, and *$P < 0.05$.
